# Types of Component Interfaces in Metal Matrix Composites on the Example of Magnesium Matrix Composites

**DOI:** 10.3390/ma14185182

**Published:** 2021-09-09

**Authors:** Katarzyna N. Braszczyńska-Malik

**Affiliations:** Faculty of Production Engineering and Materials Technology, Institute of Materials Engineering, Czestochowa University of Technology, Al. Armii Krajowej 19, 42-200 Czestochowa, Poland; kacha@wip.pcz.pl or k.braszczynska-malik@pcz.pl; Tel.: +48-34-3250-652

**Keywords:** metal matrix composite, magnesium, particles, interface, microstructure

## Abstract

In this paper, a summary of investigations of the microstructure of cast magnesium matrix composites is presented. Analyses of the interfaces between the reinforcing particles and the magnesium alloy matrices were performed. Technically pure magnesium and four various alloys with aluminum and rare earth elements (RE) were chosen as the matrix. The composites were reinforced with SiC and Ti particles, as well as hollow aluminosilicate cenospheres. Microstructure analyses were carried out by light, scanning, and transmission electron microscopy. The composites with the matrix of magnesium and magnesium–aluminum alloys with SiC and Ti particles exhibited coherent interfaces between the components. In the composites based on ternary magnesium alloy with Al and RE with Ti particles, a high-melting Al_2_RE phase nucleated on the titanium. Different types of interfaces between the components were observed in the composites based on the magnesium–rare earth elements alloy with SiC particles, in which a chemical reaction between the components caused formation of the Re_3_Si_2_ phase. Intensive chemical reactions between the components were also observed in the composites with aluminosilicate cenospheres. Additionally, the influence of coatings created on the aluminosilicate cenospheres on the bond with the magnesium matrix was presented. A scheme of the types of interfaces between the components is proposed.

## 1. Introduction

Metal matrix composites (MMCs) have been designed for many years in a variety of systems in terms of both different metal alloy matrices and various types of reinforcement phases [1,2,3,4,5,6,7,8,9,10,11,12,13,14,15,16]. In the design of composites, the selection of the main material factors is of key importance, which concerns both the chemical composition of the matrix and the chemical composition, size, and shape of the reinforcing phase. In the case of metal matrix composites, the selection of the type of matrix should be understood as the choice of the alloy itself and related alloying elements that can significantly affect the structure of the final product. The type of the reinforcing phase is important during the production of composites due to the possibility of wetting by the liquid matrix and obtaining a matrix/reinforcement bond [17,18,19,20,21,22,23,24,25,26,27,28,29]. In metal matrix composites, the choice of the chemical composition of the matrix, as well as the selection of the type of reinforcement significantly affects the possibility of shaping the designed structure, sometimes even making it impossible to produce a composite with the required level of properties [19,20,21,22,23,24,25,26,27,28,29,30,31,32,33,34,35,36,37,38,39,40]. The selection of components directly determines the types of interfaces that arise between the matrix and the material of the reinforcing phase.

In metal matrix composites, a complete lack of reaction between the components is possible. Either the so-called mechanical bond, which occurs in many metal–ceramic composites, or a bond that allows the creation of coherent interfaces between the reinforcing phase and the matrix can be distinguished here. A different type of bond between components is the creation of new phases as an additional structural component resulting from chemical reactions between the reinforcing phase and the matrix. Intensive reactions between the components may also consequently lead to changes in the phase composition of the matrix material itself [20,21,22]. Reactions between the reinforcing phase and the matrix have very often been observed in aluminum matrix composites. The most frequently observed reaction product of these composites with C_gr_ or SiC fibers or particles was the Al_4_C_3_ carbide, formed at the component interface [27,28,30,31]. Another example involves composites based on the Al–Mg-type alloy with Al_2_O_3_, in which the reaction between Mg (as the alloying element) and the reinforcement resulted in the formation of MgO or MgAl_2_O_4_, depending on the temperature and the volume fraction of Mg in the matrix alloy [28,32]. In turn, in composites based on Al–Cu and Al–Li alloys with Al_2_O_3_, CuAl_2_O_4_ and LiAlO_2_ (LiAl_5_O_8_, Li_2_O) were the products of reaction of the components, respectively [28]. The resulting bond (or lack thereof) between the reinforcing phase and the matrix directly affects the properties of the produced composites, constituting an additional structural factor.

The first research works on the types of component interfaces were created in the 1960s. In 1974, Metcalfe [24] summarized the component interfaces in metal matrix composites with fibers [25]. Based on previous work, he presented three main classes for the classification of interfaces in composites [24]:

Class I, filament and matrix mutually nonreactive and insoluble.

Class II, filament and matrix mutually nonreactive but soluble.

Class III, filament and matrix react to form compound(s) at interfaces.

Then, Metcalfe also proposed [24] six types of bonds between components, which were the mechanical bond, the dissolution and wetting bond, the oxide bond, the reaction bond, the exchange reaction bond, and the mixed bond. These works were of the first systematic examinations of types of interfaces between components. The many years of developing composite materials have also resulted in development of the production of various types of coatings on reinforcing phases. Specially produced additional layers (coatings) on reinforcing phases change the nature of the bond between the components. Coating of the reinforcement is a widely used technique that prevents reactions between the components or improves the wettability of the reinforcement by the matrix. Reinforcement coatings and interfaces in aluminum matrix composites with fibers were reviewed in detail by Rajan et al. [28]. Various types of coatings—metallic (Ni, Cu, Ti, Ag, Mo, etc.) or ceramic (SiC, SiO_2_, TiO_2_, Al_2_O_3_, etc.)—have been widely used, especially for carbon fibers. Techniques such as electroless deposition, physical (PVD) or chemical vapor deposition (CVD), the sol–gel process, or thermal spraying were used to form coatings on the reinforcement [27,28]. It should be noted, however, that both the presented early works [24,25] and often subsequent ones [26,27,28,29,30,31,32,33] did not take into account the possibility of the nucleating influence of the reinforcement on the structural components (i.e., intermetallic phases) of the metal matrix. It should be emphasized that the knowledge of the types of bonds between the matrix and the reinforcing phase and the ability to control them are key factors in shaping the structure of composites.

Due to the properties of the matrix itself, including its density, magnesium matrix composites constitute a modern group of materials, widely studied in many matrix-reinforcing phase systems. Several different magnesium matrix alloys (from the Mg–Al, Mg–Zn, or Mg–rare earth elements systems) with various reinforcing phases (various particles or fibers), for example, SiC, C_gr_, Al_2_O_3_, TiC, Ti, and fly ash microspheres etc., have been designed and investigated in recent years [1,2,3,4,8,9,10,11,12,33,34,35,36,37,38,39,40,41,42,43,44,45,46,47,48,49,50,51,52,53,54,55,56,57,58,59,60,61,62,63]. Those composites are very attractive in applications such as the aerospace, automobile, or electronics industries due to their unique combination of different properties, e.g., exceptional dimensional stability and high damping capacity, specific strength and stiffness, or resistance to abrasive wear. Magnesium matrix composites, like different metal matrix composites, can be fabricated by both various casting methods and powder metallurgy methods. In magnesium matrix composites, the interfaces between the components can be very different and also depend on both the reinforcement type and the chemical composition of the used magnesium alloy.

The article summarizes the study of the microstructure of magnesium matrix composites in order to present the different types of component interfaces. For this purpose, various types of magnesium matrix composites were selected from among those originally designed and manufactured by casting methods. The paper shows that the use of different reinforcing particles and various magnesium alloys allowed a variety of types of bonds to be obtained between the components. The analyses of some of these composites also revealed the nucleating effect of the reinforcing particles on the structural components of the matrix alloy. Additionally, the influence of additional layers formed on the reinforcing particles (i.e., coatings) on the possibility of component interfaces is presented. In the article, the description, comparison, and extensive analysis of the phenomena occurring on the component interfaces allows the presentation of many factors occurring in these materials. On the basis of the presented results of the analyses, a scheme of the types of interfaces between the components is proposed.

## 2. Materials and Methods

Four different magnesium alloys were used to produce the composites in order to reveal the influence of the alloying elements on the type of component interfaces. The chemical compositions of these alloys are presented in Table 1. The AM50 and AZ91 alloys were popular commercial magnesium alloys, while the AME505 alloy (referred to in previous works [22,64]) and the ME3 [22] double alloy were experimental alloys (made using cerium rich mischmetal with the chemical composition according to the attestation equal to 54.8 wt.% Ce, 23.8 wt.% La, 16 wt.% Nd, 5.4 wt.% Pr, 0.16 wt.% Fe, and 0.19 wt.% Mg). As the reinforcement, SiC and Ti particles, as well as hollow aluminosilicate cenospheres (called also microspheres or microballoons), were used. The SiC particles had an irregular shape and a diameter up to 40 μm. They were of mixed polymorph types 6H, 3H, and 3C. The spherical Ti particles had a fraction below 50 μm. The size distribution of the spherical aluminosilicate cenospheres was between 63 and 125 μm. The cenospheres were used in the as-received state (uncoated), after Ni–P coating by the electroless plating method (i.e., with an Ni–P amorphous layer) and after Ni–P coating and heating at 773 K (i.e., with a crystalline Ni + Ni_3_P + NiO layer). These kinds of layers were described in previous works [9,22,49,50].

For presenting the different types of component interfaces, specific magnesium matrix composites were selected and prepared using casting methods, as described below.

1. Technically pure magnesium matrix composite with SiC particles and Ti particles,
2. AM50 magnesium matrix alloy with SiC particles,
3. AME505 magnesium matrix alloy with Ti particles,
4. ME3 magnesium matrix alloy with SiC particles,
5. AZ91 magnesium matrix alloy with as-received (uncoated) aluminosilicate cenospheres,
6. AZ91 magnesium matrix alloy with Ni–P-coated aluminosilicate cenospheres and initially heated at 773 K,
7. AZ91 magnesium matrix alloy with Ni–P-coated aluminosilicate cenospheres.

Composites 1–5 were fabricated using a casting method consisting of mechanical mixing of the molten matrix alloy followed by addition of the reinforcing particles (during mixing) and gravity casting into steel molds. The composites obtained using this method, described in detail in previous works [13,22], contain up to 30 wt.% reinforcing particles in the matrix alloy. On the other hand, composites 6–7 were fabricated using the negative pressure infiltration method, in which the molten matrix alloy was poured in from the top of the mold (with loosely located aluminosilicate cenospheres). At the same time, negative pressure was applied from the bottom of the mold. Thanks to this technique (described in detail in previous works [9,22,49,50]), composites with reinforcement up to 60 vol.% in the matrix alloy could be obtained.

For the microstructure analyses, specimens from the investigated materials were prepared by standard metallographic procedures with etching in a 1% solution of HNO_3_ in C_2_H_5_OH. The magnesium alloys and composite microstructures were observed with an Olympus GX51 light microscope (LM) (Olympus, Tokyo, Japan) with differential interface contrast (DIC) and a JOEL JSM-6610LV scanning electron microscope (SEM) (JOEL Ltd., Tokyo, Japan) with an energy-dispersive X-ray spectrometer (EDX) (Oxfrod Instruments, Abingdon, UK). The interfaces between the components were also analyzed by transmission electron microscopy (TEM) by means of a Philips CM20 FEG (Philips, Amsterdam, the Netherlands) and a TECNAI G^2^ FEC (FEI Company, Hillsboro, OR, USA). For the TEM observations, thin foils from the composites were prepared using a Gatan dimple (Gatan, Pleasanton, CA, USA) and ionic Leica—EM RES 101 (Leica Microsystems GmbH, Wetzlar, Germany) equipment.

## 3. Results and Discussion

### 3.1. Coherent Interfaces between Components

**Figure 1:**
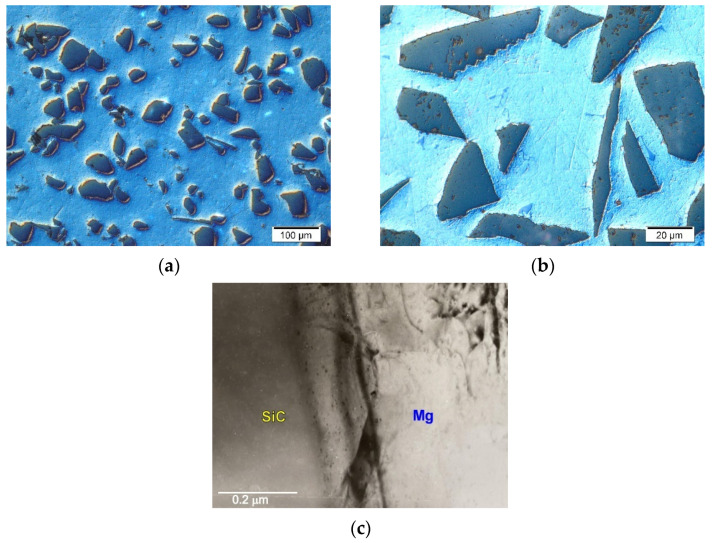
Microstructure of technically pure magnesium matrix composites with SiC particles. (**a**,**b**) Micrographs taken at different magnification; (**c**) TEM micrograph of interface between components.

In magnesium matrix composites, a kind of coherent interface of the components can occur. This type of interface is created when the reinforcing phase is wetted very well by the liquid matrix and there is no reaction between the components. It can be observed in Mg–SiC or Mg–Ti systems. Figure 1 shows exemplary micrographs of the microstructure of the composite made on the matrix of technically pure magnesium with SiC particles. Silicon carbide is highly wettable by liquid magnesium, while showing a complete lack of reactivity with magnesium [1,10,12,13,22,34,35,36,37,40,41,42,43,44]. A characteristic feature of this type of system of components is the formation of coherent interfaces between the SiC particles and magnesium.

These interfaces often show a high degree of correlation of crystallographic orientations between the components. For SiC particles with the 6H polymorph, for example, the relationships of crystallographic orientations with a matrix of the following types were determined [41,42]: [$\bar{2}$113]_Mg_//[10$\bar{1}$0]_SiC_, (10$\bar{1}$1)_Mg_//(0006)_SiC_, ($\bar{2}$20$\bar{2}$)_Mg_//(1$\bar{2}$1$\bar{6}$)_SiC_ and [1$\bar{1}$00]_Mg_//[01$\bar{1}$0]_SiC_, (0002)_Mg_//(0006)_SiC_, (11$\bar{2}$0)_Mg_//($\bar{2}$110)_SiC_. Nonetheless, attention should be paid to the presence of silicon carbide in many polymorphs, which directly affects the possibility of the formation of specific dependencies of crystallographic orientations. The most commonly used in composites is hexagonal SiC 6H or a mixture of 6H, 3H, and 3C particles [22,52]. Moreover, the alloying elements dissolved in magnesium influence the parameters of the structure of magnesium itself in different ways. For instance, aluminum dissolved in magnesium causes significant contraction of its lattice parameters. However, obtaining low-energy interfaces of the components directly determines the properties of the produced composites.

Some research papers [1,13,22,41,42,43] considered the nucleating effect of silicon carbide particles during the crystallization of magnesium due to the aforementioned relations of crystallographic orientation on the Mg–SiC interface. It should be noted, however, that the heterogeneous nucleation of metal on solid particles depends not only on the possibility of creating coherent interfaces between the substrate and the nucleating metal, but also on other factors such as the size and shape of the particles (taking into account in the analysis of the solidification modulus) or the temperature gradient during solidification [22].

**Figure 2:**
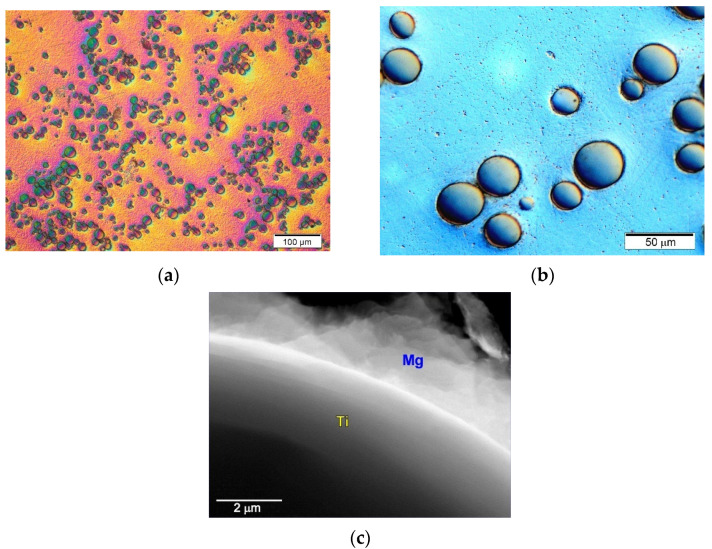
Microstructure of technically pure magnesium matrix composites with Ti particles. (**a**,**b**) Micrographs taken at different magnification; (**c**) TEM micrograph of interface between components.

Another type of magnesium composites that produce coherent interfaces between the components are composites with Ti or Ti6Al4V particles.These composites are often produced by powder metallurgy methods, mainly with particles of irregular morphology [2,3,11,53,54,55,56], but it is also possible to produce them by casting methods [22,57,59]. The metallic reinforcing phase shows very good wettability by the liquid matrix (in the absence of the solubility of Ti in Mg and the absence of intermetallic phases between Mg and Ti). In the wettability test by the sessile drop method, the contact angle of titanium with magnesium was determined to be 31° at the temperature of 1073 K after 180 s [58]. Sample micrographs of the microstructure of the composite on a magnesium matrix with spherical Ti particles are presented in Figure 2.

The interfaces between the components in the cast Mg–Ti materials show a permanent and coherent bond, without the presence of microporosity observed at the Mg/Ti interfaces in the composites produced by powder metallurgy [53,54,55,56]. Moreover, due to the hexagonal lattice of both metals, it is possible to correlate the crystallographic orientations between them. It should be noted that, in the casting method used in this work, the slurry preparation and casting process were carried out below the titanium allotropic transformation temperature, amounting to 1155 K. According to the basic theoretical calculations in the directions of the magnesium and titanium matrix plane, the degree of mismatch of the lattice constants is 0.08, which is below the critical value for the formation of coherent interfacial interfaces.

#### Eutectics Distributed During Solidification

**Figure 3:**
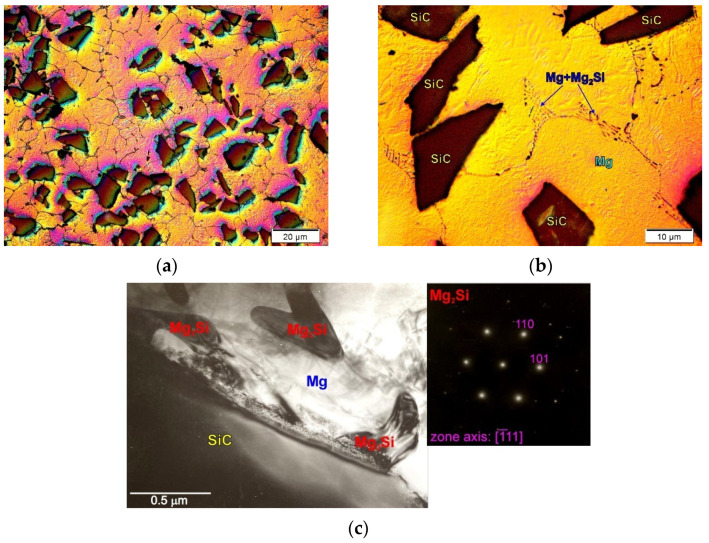
Microstructure of magnesium matrix composites with SiC particles. (**a**,**b**) Micrographs taken at different magnification; (**c**) TEM micrograph of interface between components with diffraction pattern from observed Mg_2_Si phase.

When analyzing composites with coherent component interfaces, attention should be paid to possible oxidation processes of the reinforcing phase. Reinforcing phases (for example, SiC) stored in a loose form may undergo natural surface oxidation at ambient temperature. Although silicon carbide is very stable in magnesium, it is very often coated with SiO_2_ as a result of surface oxidation [22,34,37,43]. During the production of the composite slurry, magnesium reduces the silicon oxide. Silicon introduced in this way to magnesium changes the chemical composition of the matrix, causing the formation of a fully divorced Mg + Mg_2_Si eutectic during solidification. The presence of significant volume fractions of this eutectic, mainly in the interdendritic regions, is easily observed in composites produced especially on the basis of technically pure magnesium. Figure 3 shows examples of the microstructure of the cast composite on a technically pure magnesium matrix.

In this case, the Mg_2_Si compound was formed not due to the reaction of Mg with SiC, but as a result of the reaction of Mg with SiO_2_ originally present on the SiC particles. Silicon has a very slight (even negligible) solubility in magnesium (equal only to 0.003 wt.% at the temperature of eutectic transformation—911.9 K). For this reason, during the non-equilibrium solidification of Mg_2_Si and practically pure Mg, a eutectic mixture was formed. The distribution of the eutectic was in turn the result of nonequilibrium solidification of the composite matrix. In this case, the sometimes-observed presence of the Mg_2_Si compound in the SiC particles is, therefore, the consequence of the matrix solidification process, and not the reaction between magnesium and SiC.

At this point, it should also be noted that the same microstructure of the composite presented in Figure 3 can be obtained in two ways: the one presented above (by introducing oxidized SiC particles into the magnesium matrix) or by introducing non-oxidized SiC particles into the hypereutectic Mg–Si alloy (described in previous works [22,52]). Additionally, due to the properties of the Mg_2_Si phase, materials from Mg–Si system are often called composites [22,45,46,51]. According to this nomenclature, the above Mg–Mg_2_Si–SiC composite is an example of a “mixed” type composite due to the production method in which the SiC particles are introduced from the outside (ex situ), while Mg_2_Si is formed inside the suspension (in situ).

**Figure 4:**
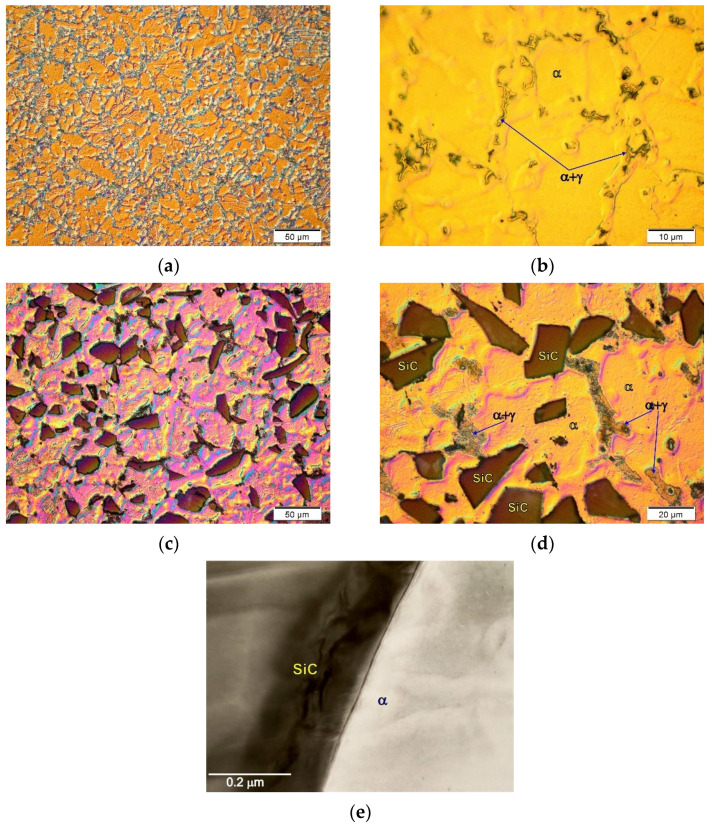
Microstructure of AM50 magnesium alloy. (**a**,**b**) Micrographs taken at different magnification) and microstructure of AM50 matrix composites with SiC particles. (**c**,**d**) Micrographs taken at different magnification; (**e**) TEM micrograph of interface between components.

Composites based on technically pure magnesium are a reference point for analysis of the influence of alloying elements on the possibilities of creating bonds between the components. Nevertheless, they are of little practical importance due to the low properties of magnesium itself. Therefore, composites are most often produced on a matrix of magnesium alloys from the Mg–Al, Mg–Zn, or Mg–RE systems [22]. For composites based on Mg–Al alloys reinforced with SiC particles, no negative influence of aluminum on the shaping of the material structure or the bond between the components was noted [22,43]. Sample micrographs of the microstructure of the matrix alloy itself and the composite on the matrix of the AM50 alloy with SiC particles are presented in Figure 4.

The microstructure of the AM50 matrix alloy consisted of a solution of solid aluminum in magnesium α(Mg) and eutectic α + γ (where γ is the Mg_17_Al_12_ intermetallic compound) formed as a result of non-equilibrium solidification. In this alloy (as in the AZ91 alloy), the presence of the Al_8_Mn_5_ intermetallic compound is also observed [18,22]. In the composite based on the AM50 alloy, the interfaces between the SiC particles and the matrix, as in the case of the composites based on pure magnesium, show a similar character of the cohesive bond. It should also be emphasized that the sometimes-observed local increase in aluminum concentration at the component interface or the presence of eutectic α + γ was the result of the segregation of the alloying elements in the matrix alloy itself during its non-equilibrium solidification, and not the interaction between the components. In metal composites containing aluminum in the matrix and carbon in the reinforcing phase, formation of the unfavorable Al4C3-type carbide is possible, as often observed in aluminum composites [7,19,24,28]. However, due to the low concentration of aluminum dissolved in magnesium, no reaction effects leading to the formation of carbide phases in the composites with a matrix of Mg–Al alloys are observed. It should also be added that coherent component bonds were also observed for the composites produced on the different hypoeutectic alloys such as Mg–Zn and Mg–Zn–Zr with SiC, presented in previous works [22,43,47].

### 3.2. Nucleation of Matrix Phases on Reinforcement

**Figure 5:**
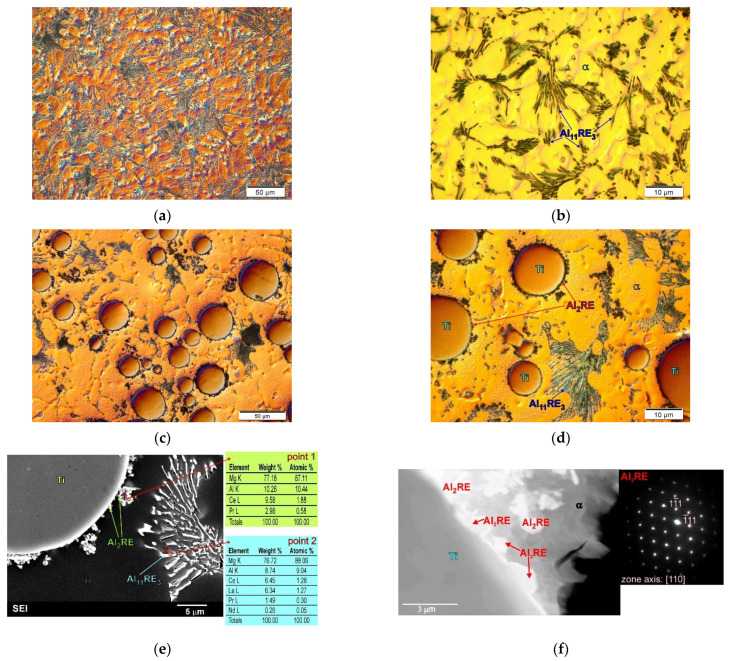
Microstructure of AME505 magnesium alloy. (**a**,**b**) Micrographs taken at different magnification and microstructure of AME505 matrix composites with SiC particles. (**c**,**d**) Micrographs taken at different magnification; (**e**) SEM micrograph of composite microstructure with EDX results from designated points; (**f**) TEM micrograph of interface between components with diffraction pattern from observed Al_2_RE phase.

On the other hand, a different phenomenon occurred after introducing Ti particles into the alloys of magnesium with aluminum and rare earth elements (AME505). Figure 5 shows examples of the microstructure micrographs of both the unreinforced AME505 alloy and the composite based on the AME505 alloy matrix with Ti particles. Figure 5c–f show the Al_2_RE phase distributed at the component interfaces.

Ti particles do not react with the alloying elements present in AME505 either [22,62]. Nonetheless, the microstructural analyses revealed a significant influence of titanium on the nucleation of the high-melting Al_2_RE phase. As the theoretical calculations show, although the Al_2_RE phase has a regular structure, it is possible to create coherent interfaces with titanium in the basal planes of both phases in the directions <110> of the Al_2_RE and <11$\bar{2}$0> of the Ti phases. It should be emphasized that, in this case, the introduction of Ti particles into the AME505 alloy does not result in a qualitative change in the phase composition of the matrix alloy, but in a significant difference in the volume fraction of individual structural components. In the microstructure of the cast AME505 alloy, the Al_2_RE phase occurs with a small volume fraction, practically not allowing observations using light microscopy or scanning electron microscopy [22,64]. The main structural components of the AME505 alloy are the α-Mg solid solution and the Al_11_RE_3_ intermetallic phase with acicular morphology (Figure 5a,b).

In the composite based on the AME505 alloy with Ti particles, the Al_2_RE phase nucleated on the Ti particles, which caused a significant increase in its volume fraction and, thus, a decrease in the volume fraction of the next phase, i.e., Al_11_RE_3_ (Figure 5c–f). Changes in the volume fraction of the phases in the matrix of the composites make it difficult to compare the behavior of the composites with the matrix alloy itself. It should also be noted that the presented phenomenon of nucleation of the intermetallic phases of the matrix on the reinforcing phase was also revealed in the composite based on the matrix of the AM50 alloy with Ti particles presented in previous work [59]. In this case, the Al_8_Mn_5_ phase nucleated on the Ti particles. In contrast to the composite presented above (AME505-Ti), in materials based on the AM50 alloy matrix, nucleation of the intermetallic phase did not change the phase composition of the matrix alloy. The volume fraction of the Al_8_Mn_5_ phase was small and constant in both the Mg–Al alloys and composites on their matrix. It should be emphasized that, in the described examples, the intermetallic phases present at the component interface do not arise as a result of chemical reactions between the reinforcing phase and the matrix, but as a result of their nucleation on the reinforcement.

### 3.3. Reactions between Reinforcement and Matrix Alloy

Reactions between components are most often detrimental due to degradation of the reinforcing phase and the formation of brittle phases at the interface. Reactions between the reinforcing phase and the matrix have very often been observed in aluminum composites [27,28,30,31]. Using the example of the magnesium composites presented below, two types of reactions can be distinguished. The first is when a chemical reaction takes place between the reinforcement and the alloying elements, and the second is when the reinforcing phase reacts directly with the main matrix metal (i.e., magnesium).

**Figure 6:**
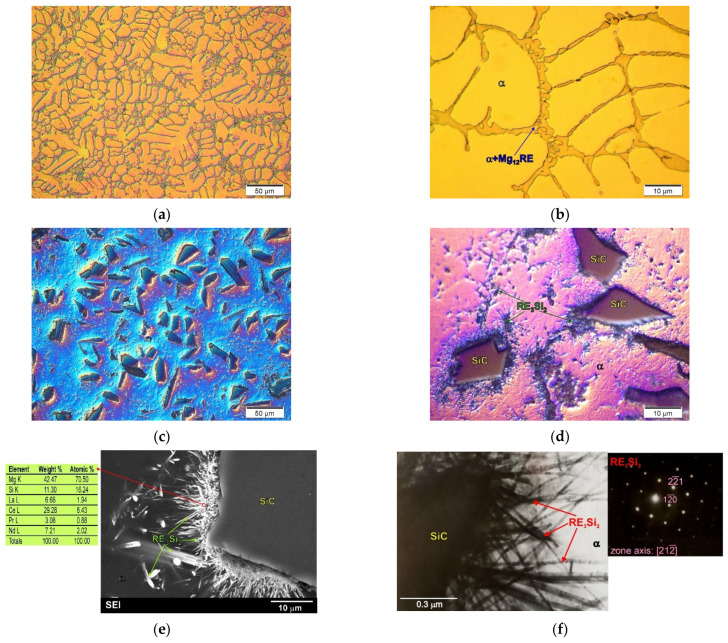
Microstructure of ME3 magnesium alloy. (**a**,**b**) Micrographs taken at different magnification) and microstructure of ME3 matrix composites with SiC particles. (**c**,**d**) Micrographs taken at different magnification); (**e**) SEM micrograph of composite microstructure with EDX results from designated point; (**f**) TEM micrograph of interface between components with diffraction pattern from observed RE_3_Si_2_ phase.

In magnesium matrix composites, an intense reaction between the components can be observed in materials based on Mg–RE-type alloys with SiC [22,38,43]. Figure 6 shows the micrographs of the microstructure of the ME3 alloy and the composite on the matrix of this alloy with SiC particles. The microstructure of the unreinforced ME3 matrix alloy consisted of a solid solution of rare earth elements in magnesium (α phase) and the α + Mg_12_RE eutectic. It should be noted that detailed microstructural analyses of the composite produced on the basis of this alloy revealed an intense reaction between the rare earth elements dissolved in magnesium and the silicon carbide particles. This reaction consequently led to the formation of what can be generally referred to as the RE_3_Si_2_ phase. This compound, formed at the interface of the components, has an acicular morphology, which is unfavorable due to the properties of composites. Additionally, the SEM and TEM micrographs of the component interface, attached in Figure 6e,f, clearly show the precipitate of the RE_3_Si_2_ phase formed on the SiC particles. The intense chemical reaction between SiC and rare earth elements present in the matrix alloy led to degradation of the reinforcement. Due to the chemical reaction of the components and mechanical mixing during the production of the composites, the RE_3_Si_2_ phase also forms inside the matrix material. Thus, the introduction of SiC particles to the liquid Mg–RE alloy resulted in an intense reaction between the components, leading to the formation of a new phase in the microstructure of the composite.

**Figure 7:**
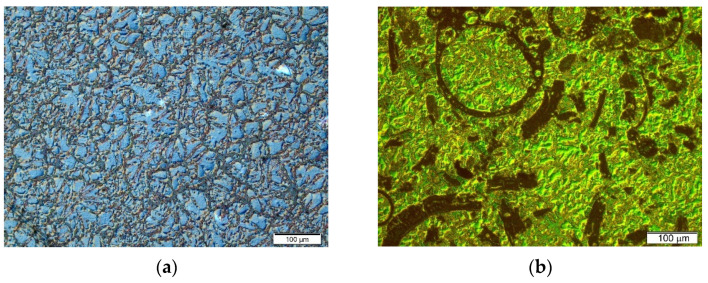
Microstructure of AZ91 magnesium alloy (**a**) and AZ91 matrix composite with as-received (uncoated) aluminosilicate cenospheres (**b**).

Another example of a material in which intense reactions occur between the components are composites based on magnesium with aluminosilicate cenospheres. Microspheres are most widely used as a component for aluminum matrix composites, although research has also been conducted for a Pb or Fe matrix [5,14,22]. Notwithstanding, the main difficulty of their application in the production of magnesium matrix composites is the strong reactivity of magnesium with the oxides forming the walls of the microspheres. The reduction of oxides by liquid magnesium is highly exothermic. Regardless of the production method used, the walls of the microspheres crack as a result of the chemical reaction and are filled with the liquid matrix. In this case, the reaction occurs between the magnesium itself (and not the alloying elements) and the cenospheres. The reduction of the oxides by magnesium also results in the formation of large volume fractions of the Mg_2_Si and MgO phase inside the composite material. An example of a typical structure of a magnesium composite based on the AZ91 alloy with aluminosilicate microspheres produced by gravity casting of a mechanically stirred suspension is shown in Figure 7.

In recent years, many research works have been concerned with the production of magnesium composites with aluminosilicate microspheres, in which a different mutual volume fraction of undamaged microspheres to fractured, matrix-filled microspheres was obtained [4,8,9,14,22,63]. It should be emphasized that the materials with the microstructure shown in Figure 7 can be treated as hybrid composites of the Mg–microsphere walls–MgO–Mg_2_Si type. They are also an example of a “mixed” type composite due to the production method in which the microspheres are introduced from the outside (ex situ), while MgO and Mg_2_Si are formed inside the suspension (in situ). Such materials are characterized, among others, by an increase in hardness or abrasion resistance; nonetheless, they do not achieve the reduction in the density of the manufactured parts, as assumed at the design stage, in relation to the matrix material.

### 3.4. Additional Layer (Coating) on Reinforcement

**Figure 8:**
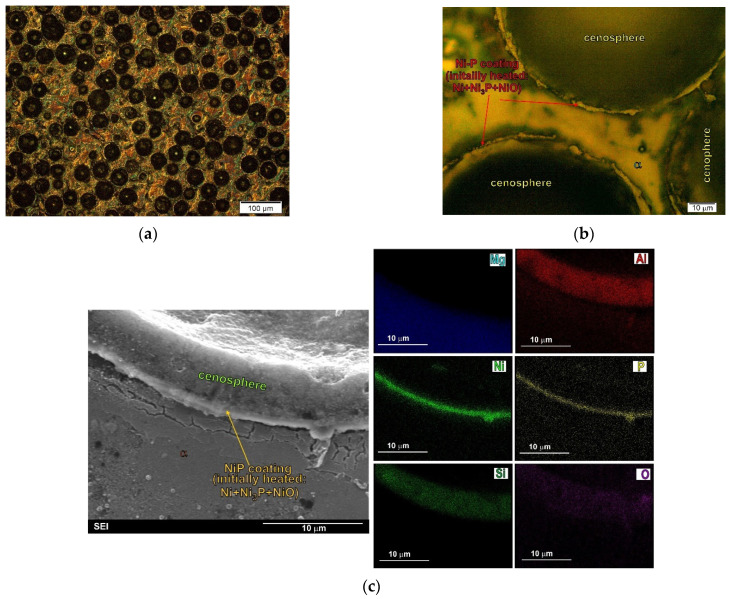
Microstructure of AZ91 magnesium matrix composites with Ni–P-coated (and initially heated) aluminosilicate cenospheres. (**a**,**b**) Micrographs taken at different magnification); (**c**) SEM micrograph of composite microstructure with EDX results.

A way to obtain magnesium composites with uncracked aluminosilicate cenospheres is to use layers of an additional material covering the microspheres and protecting them from direct contact with the liquid magnesium. The application of the electroless deposition method in the case of spherical fly ash seems to be the most effective. This solution enables the formation of layers, e.g., of the Ni–P type, on the aluminosilicate microspheres. Due to the provision of the most favorable conditions in the process of vacuum infiltration, the most effective is also the use of preheated microspheres with an Ni–P layer, which was described in previous study [9,22,50]. Examples of micrographs of the microstructure and interface between the components in the composite made on the basis of the AZ91 alloy with coated (Ni–P) and preheated aluminosilicate cenospheres are presented in Figure 8.

As a result of this heat treatment performed in air atmosphere at 773 K, the amorphous Ni–P layer (formed during the electroless deposition method) crystallizes to form fine crystalline Ni and Ni_3_P compounds inside the layer itself, as well as NiO on its surface as a result of oxidation. The use of such coated microspheres for the production of composites by the method of vacuum infiltration facilitates the technological process due to the change in liquid magnesium wettability of the NiO layer in relation to Ni–P [22]. In the microstructure of the composites produced using microspheres covered with a protective layer and annealed, one observes on the particles successively an Ni–P crystal layer (which is a material composed of fine crystalline Ni and Ni_3_P) and an NiO layer on the border of contact with the magnesium matrix, which was described in previous works [49,50]. This type of coating is stable during the production of composites and does not react with the liquid matrix alloy. Figure 8c presents SEM micrograph of composite microstructure with EDX results, which demonstrate a continuous layer between the cenospheres and the matrix alloy. The created protective layer (coating) protected the walls of the cenospheres against reaction with magnesium and allowed a composite with unbroken cenospheres to be obtained. The research on this type of composites produced on the basis of AZ91 and AM50 alloys [22,50] did not reveal the nucleating effect of this coating on the intermetallic phases present in the matrix alloy.

### 3.5. Reactions between Additional Layer (Coating) on Reinforcement and Matrix Alloy

**Figure 9:**
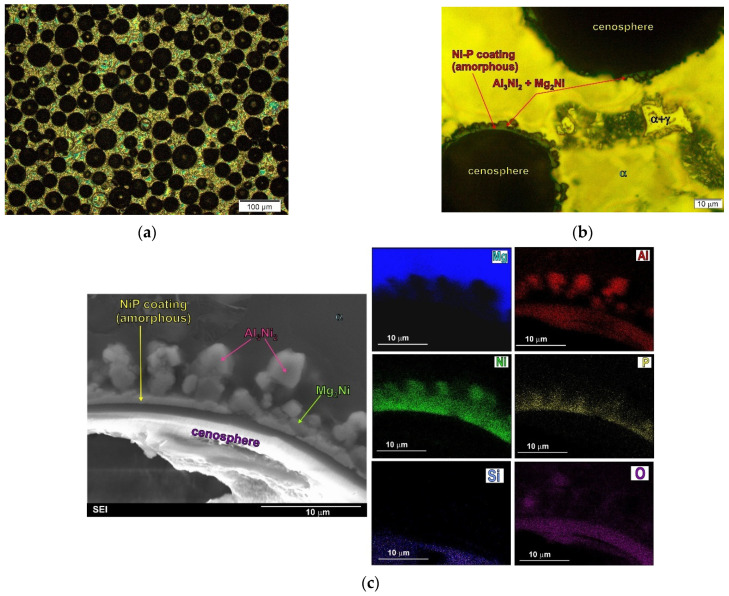
Microstructure of AZ91 magnesium matrix composites with Ni-P coated aluminosilicate cenospheres. (**a**,**b**) Micrographs taken at different magnification; (**c**) SEM micrograph of composite microstructure with EXD results.

However, a different kind of interface is obtained by using the aluminosilicate cenospheres described above, covered with an Ni–P layer, although not subjected to preliminary heat treatment (i.e., annealing). Figure 9 shows the micrographs of the microstructure and the interface in the composite made on the base of the AZ91 alloy with aluminosilicate cenospheres, covered with an amorphous Ni–P layer.

Analyses of the influence of the Ni–P layer on the shaping of the microstructure of magnesium composites showed that the amorphous layer obtained in the process of electroless deposition may react with liquid magnesium, as a result of which the Mg_2_Ni compound is formed [22]. When magnesium–aluminium alloys are used in the matrix, the reaction between the amorphous Ni–P layer and the liquid matrix also leads to the formation of Al_3_Ni_2_ phases. Both intermetallic compounds were observed at the component interface (between the coating layer and the matrix alloy) [9,22,49,50]. In the case of the obtained composite, despite the reaction between the coating and the matrix alloy, the formed protective layer (Ni–P) prevents contact between the magnesium and the aluminosilicate cenospheres walls and also prevents cracking of the cenospheres. Nevertheless, the type of interface in this case is different than for the composites made with the preheated Ni–P-coated cenospheres. The compounds formed as a result of the reaction of the coating with the matrix alloy were also observed in aluminum composites for which Ni-coated or Cu-coated carbon or graphite fibers were used [28]. During fabrication, reactions between the matrix and the coatings formed NiAl_3_ and CuAl_2_ phases, respectively, at the component interfaces (around the fibers). In both cases, these reaction products also prevented the carbon fiber from reacting with the aluminum matrix (reinforcement degradation and Al_4_C_3_ carbide formation). The chemical reactions between the coating and the matrix alloys, therefore, change the chemical and phase composition of the composites, resulting in the formation of new phases.

It should also be added that, in the case of using eutectic magnesium alloys (for example, the AM50 or AZ91 alloy) as composite matrices with coated cenospheres (heated or nonheated), the sometimes-observed presence of eutectic near the component interfaces was the result of segregation of the alloying elements in the matrix alloy itself during its non-equilibrium solidification, and not the interaction between the components [9,22,50].

## 4. Summary

**Figure 10:**
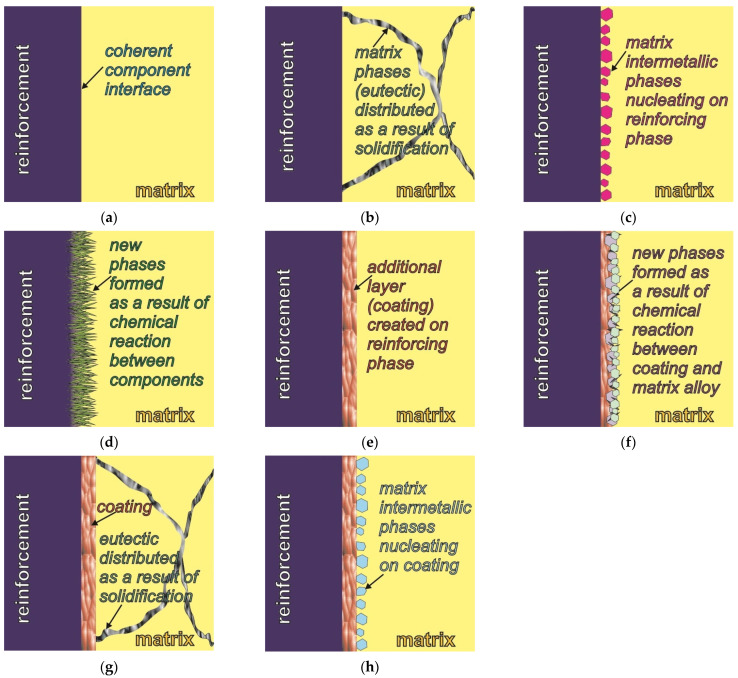
Scheme of types of interfaces between components in metal matrix composites: (**a**) coherent interface between components; (**b**) matrix phases (eutectics) distributed at component interface during solidification; (**c**) matrix phases (intermetallic) nucleated at reinforcement; (**d**) new phases at component interface forming due to reaction between components; (**e**) stable coating initially created on reinforcement; (**f**) new phases at component interface formed due to reaction between matrix alloy and coating layer; (**g**) matrix phases (eutectics) distributed during solidification; (**h**) matrix phases nucleated on coating.

The examples of magnesium matrix composites with various particles in this article illustrate the different types of bonds between the components. They take into account the coherent interface between the components, reactions between the components, and the influence of the coating of the reinforcing phase on the creation of various interfaces with the matrix. The above-described types of interfaces between the components are presented schematically in Figure 10. The proposed scheme additionally takes into account the presence of eutectic phases distributed as a result of the solidification of matrix alloys, as well as the nucleating effect of the reinforcement on the structural components of the matrix alloys (i.e., intermetallic phases which are not formed as a result of reactions between the components but nucleate on the reinforcing phase). Usually, in the case of magnesium matrix composites in which there is a coherent bond between the components, shown schematically in Figure 10a,b, the primary α-Mg phase is nucleated on the reinforcing phase. The primary α-Mg phase can also nucleated on the coating (Figure 10e,g). On the other hand, when new phases form as a result of a chemical reaction between the components (Figure 10d,f) or when matrix intermetallic phases nucleate on the reinforcing phase (Figure 10c), the phase composition of the matrix can change.

It should also be noted that when analysing the possibilities of creating a bond between components in composites, it should be presumed that there is also the phenomenon of nucleation of the intermetallic phases that are structural components of the matrix on the coating layers formed on the reinforcing particles. Such an effect of the coating (i.e. nucleating for the intermetallic phases of the matrix) is hypothetically possible, which is shown schematically in Figure 10h. Notwithstanding, in the various magnesium composites produced so far in presented research, this type of influence has not been revealed to date. 

## Figures and Tables

**Table 1:** Nominal chemical composition of used magnesium matrix alloy (where RE denotes a rare earth elements) [22].

Alloy	Chemical Composition wt%				
	Al	Zn	Mn	RE	Mg
AM50	4.9	-	0.26	-	rest
AZ91	8.7	0.13	0.7	-	rest
AME505	5.0	-	0.26	5.0	rest
ME3	-	-	-	3.0	rest
